# Accuracy of molecular biology techniques for the diagnosis of *Strongyloides stercoralis* infection—A systematic review and meta-analysis

**DOI:** 10.1371/journal.pntd.0006229

**Published:** 2018-02-09

**Authors:** Dora Buonfrate, Ana Requena-Mendez, Andrea Angheben, Michela Cinquini, Mario Cruciani, Andrea Fittipaldo, Giovanni Giorli, Federico Gobbi, Chiara Piubelli, Zeno Bisoffi

**Affiliations:** 1 Centre for Tropical Diseases, Sacro Cuore–Don Calabria Hospital, Negrar, Verona, Italy; 2 Barcelona Institute for Global Health, ISGlobal-CRESIB, Universitat de Barcelona, Spain; 3 IRCCS-Istituto di Ricerche Farmacologiche Mario Negri, Milan, Italy; 4 Centre of Community Medicine and Infectious Diseases Service, ULSS 9 Scaligera, Verona, Italy; University of Pennsylvania, UNITED STATES

## Abstract

**Background:**

*Strongyloides stercoralis* infection is a neglected tropical disease which can lead to severe symptoms and even death in immunosuppressed people. Unfortunately, its diagnosis is hampered by the lack of a gold standard, as the sensitivity of traditional parasitological tests (including microscopic examination of stool samples and coproculture) is low. Hence, alternative diagnostic methods, such as molecular biology techniques (mostly polymerase chain reaction, PCR) have been implemented. However, there are discrepancies in the reported accuracy of PCR.

**Methodology:**

A systematic review with meta-analysis was conducted in order to evaluate the accuracy of PCR for the diagnosis of *S*. *stercoralis* infection. The protocol was registered with PROSPERO International Prospective Register of Systematic Reviews (record: CRD42016054298). Fourteen studies, 12 of which evaluating real-time PCR, were included in the analysis. The specificity of the techniques resulted high (ranging from 93 to 95%, according to the reference test(s) used). When all molecular techniques were compared to parasitological methods, the sensitivity of PCR was assessed at 71.8% (95% CI 52.2–85.5), that decreased to 61.8% (95% CI 42.0–78.4) when serology was added among the reference tests. Similarly, sensitivity of real-time PCR resulted 64.4% (95% CI 46.2–77.7) when compared to parasitological methods only, 56.5% (95% CI 39.2–72.4) including serology.

**Conclusions:**

PCR might not be suitable for screening purpose, whereas it might have a role as a confirmatory test.

## Author summary

*Strongyloides stercoralis* is a worm that can potentially kill affected immunosuppressed individuals. The parasite is not easily detected with stool microscopy, hence novel tests have been implemented, such as polymerase chain reaction (PCR), that can detect the DNA of the parasite. This work aims at evaluating the performance of PCR for the diagnosis of *S*. s*tercoralis* through a revision of diagnostic studies available in literature. According to the results of this work, PCR for *S*. *stercoralis* still misses a relevant proportion of infected people, while a positive result confirms the presence of the infection.

## Introduction

*Strongyloides stercoralis* is a soil-transmitted helminth (STH) affecting around 370 million people worldwide, particularly in remote rural areas[1].

Chronic strongyloidiasis is characterized by non-specific, mostly mild symptoms involving the gastrointestinal tract (abdominal pain, diarrhea), the respiratory system (symptoms resembling asthma, chronic obstructive pulmonary disease), the skin (pruritus, rash)[2]. However, in immunosuppressed individuals the infection can become severe, with complications due to a heavier load of parasites, including intestinal obstruction, paralytic ileus, respiratory failure, death [2]. Hence it is recommended to diagnose and treat strongyloidiasis when still in the chronic, indolent phase. First-line treatment is with ivermectin, which demonstrated a good safety profile and is highly effective for chronic infection [3]. On the other hand, treatment of the severe syndrome is more complicated as failures tend to occur with the standard regimens [4].

A gold standard for the diagnosis of strongyloidiasis is still lacking [5]. Microscopic examination of stools has insufficient sensitivity, and enrichment techniques (Ritchie's method, for instance) and examination of multiple samples can only partially improve the performance of the method[6]. The Baermann method has a sensitivity about four times higher than formol-ether concentration technique (FECT); however, it is a cumbersome method and the sensitivity remains not adequate, either [5,6]. Sensitivity of agar plate culture (APC–and in particular, the technique described by Koga) is comparable to the one demonstrated by Baermann.[5] There are different serological tests, some of which are commercially available. Globally, serology demonstrated high sensitivity (ranging from 70 to 95%, depending on the test, according to a diagnostic study on multiple serological tests)[7], but there are concerns about its specificity, because of possible cross-reactions with other parasites and long-term persistence of antibodies after an effective treatment. A recombinant antigen (NIE) has been used in order to increase specificity of serological methods such as ELISA and luciferase immunoprecipitation system (LIPS) [8,9].

The latter test (NIE-LIPS) in particular demonstrated a high specificity and an equivalent sensitivity compared with the other serological tests, but the technique at the moment is not commercially available, and has been used so far only for study purpose [7].

Molecular methods have been implemented in this context, with the aim to achieve the highest sensitivity, preserving a high specificity. However, different studies report either better [10] or worse [11] accuracy of molecular methods compared to other fecal-based methods. Some variables (such as setting in which the research was conducted, population, type of molecular technique, comparator) might influence the global evaluation of their accuracy. In conclusion, the accuracy of molecular biology techniques for the diagnosis of *S*. *stercoralis* infection should be better defined, and so should their role in different settings.

Aim of this work was to review the accuracy of molecular biology techniques for the diagnosis of *S*. *stercoralis* infection.

## Methods

The protocol was registered with PROSPERO International Prospective Register of Systematic Reviews (record: CRD42016054298) on December 29^th^, 2016.

### Search strategy and selection criteria

A systematic literature search was carried out on January 20^th^, 2017. The following databases were searched for relevant studies:

- Cochrane Central Register of Controlled Trials (CENTRAL 2017, Issue 1);
- MEDLINE (PubMed) (1966 to 20 January 2017);
- EMBASE (Embase.com) (1974 to 20 January 2017);
- Latin American and Caribbean Health Science Information Database (LILACS) (Bireme) (1982 to 20 January 2017);
- ClinicalTrials.gov
- World Health Organization (WHO) International Clinical Trials Registry Platform

All relevant studies were reviewed, regardless of language or publication status (published, unpublished, in press, and ongoing). The reference lists of all included studies for other potentially relevant research and authors’ personal collections (grey literature) were also reviewed.

### Selection of studies

**Inclusion criteria**:

- Cohort studies
- All studies evaluating a molecular biology technique:either conventional polymerase chain reaction (PCR), nested PCR, real-time PCR (qPCR), or loop-mediated isothermal amplification (LAMP) in comparison to serology and/or fecal-based methods “specific” for the diagnosis of *S*. *stercoralis* infection (Baermann method, agar plate culture, Harada-Mori culture, combination of fecal methods).
- Studies that pooled multiple intestinal parasites into one outcome measure (for example, multiplex PCR including other soil-transmitted helminthes) were included when it was possible to disaggregate the data.
- Studies conducted in endemic as well as in non-endemic areas.
- Studies conducted on either immunocompetent or immunosuppressed patients.

**Exclusion criteria**:

- Case-control studies
- Non-human studies
- Duplicate publications

Two authors, DB and ARM, reviewed the titles and abstracts yielded by the search, and identified all studies that potentially met the inclusion criteria. DB contacted some authors requesting additional information on published data and/or other potentially relevant unpublished data. After obtainment of the full text articles of the records selected as potentially relevant, DB and ARM independently assessed whether or not each study met the inclusion criteria using an eligibility form in Excel. When DB and ARM did not reach a consensus, a third reviewer (AA) made the final inclusion decision.

### Data collection process

DB and ARM independently performed the data extraction, that included sensitivity and specificity values, and other covariates, namely: reference test (divided in four categories: serology, culture, Baermann, combination of parasitological exams), setting (endemic/non endemic area), population (children, adults, all ages, not specified), immunological status (immunocompetent, immunosuppressed, not specified). For study purpose, infected and not infected were all subjects resulting positive and negative, respectively, to the reference standard test(s). The sensitivity of the index test(s) was calculated as the proportion of true positives (positive at the index test over all infected), and the specificity as the proportion of true negatives (negative at the index test over all not-infected). Studies evaluating more than one molecular method or using more than one reference standard test were split into sub-studies. Any disagreements regarding the data extraction was solved by discussion between the two authors. When necessary, a third review author (AA) facilitated the discussion until consensus was reached.

### Risk of bias (quality) assessment

DB and ARM independently assessed the methodological quality of each included study using the QUADAS- 2 tool[12]. Hence, four key domains were evaluated in terms of risk of bias: patient selection, index test, reference standard, flow and timing. When necessary, a third review author, AA, facilitated discussion until consensus was reached. All assessments were summarized in 'Risk of bias' tables.

### Statistical analysis

The values of sensitivity and specificity were automatically computed in RevMan 2014 (Version 5.3[13]). Individual study results were graphically expressed by plotting the estimates of sensitivity and specificity and their 95% confidence intervals (CIs) through both forest plots and receiver operating characteristics (ROC) space. Heterogeneity was firstly evaluated by inspecting forest plots to detect overlapping 95% CIs, then by using a bivariate random-effects model[14] to obtain estimates of the between-study variation in sensitivity and specificity and the correlation between the two. The same bivariate model was used to assess the operating point sensitivity and specificity of the diagnostic tests under scrutiny, together with likelihood ratios and summary diagnostic odds ratio (DOR), taking both heterogeneity and threshold effect into account. Also, for each study, we estimated the true prevalence using the apparent prevalence, test sensitivity and specificity, as described by Rogan and Gladen[15]. Finally, we used the hierarchical summary ROC (HSROC) model [16] to obtain an adjusted ROC curve that summarized the results of all studies. All analyses were performed using all articles first, then they were repeated considering only those with parasitological methods (defined as the use of either stool culture, Baermann, or a combination of the two) as the reference test. This was considered the primary analysis. In order to have a more precise estimate of the influence of the real-time PCR, we also conducted a secondary analysis repeating the primary only on studies that used real-time PCR as the index test. All analyses were performed using Stata IC 13.0.

## Results

**Fig 1:**
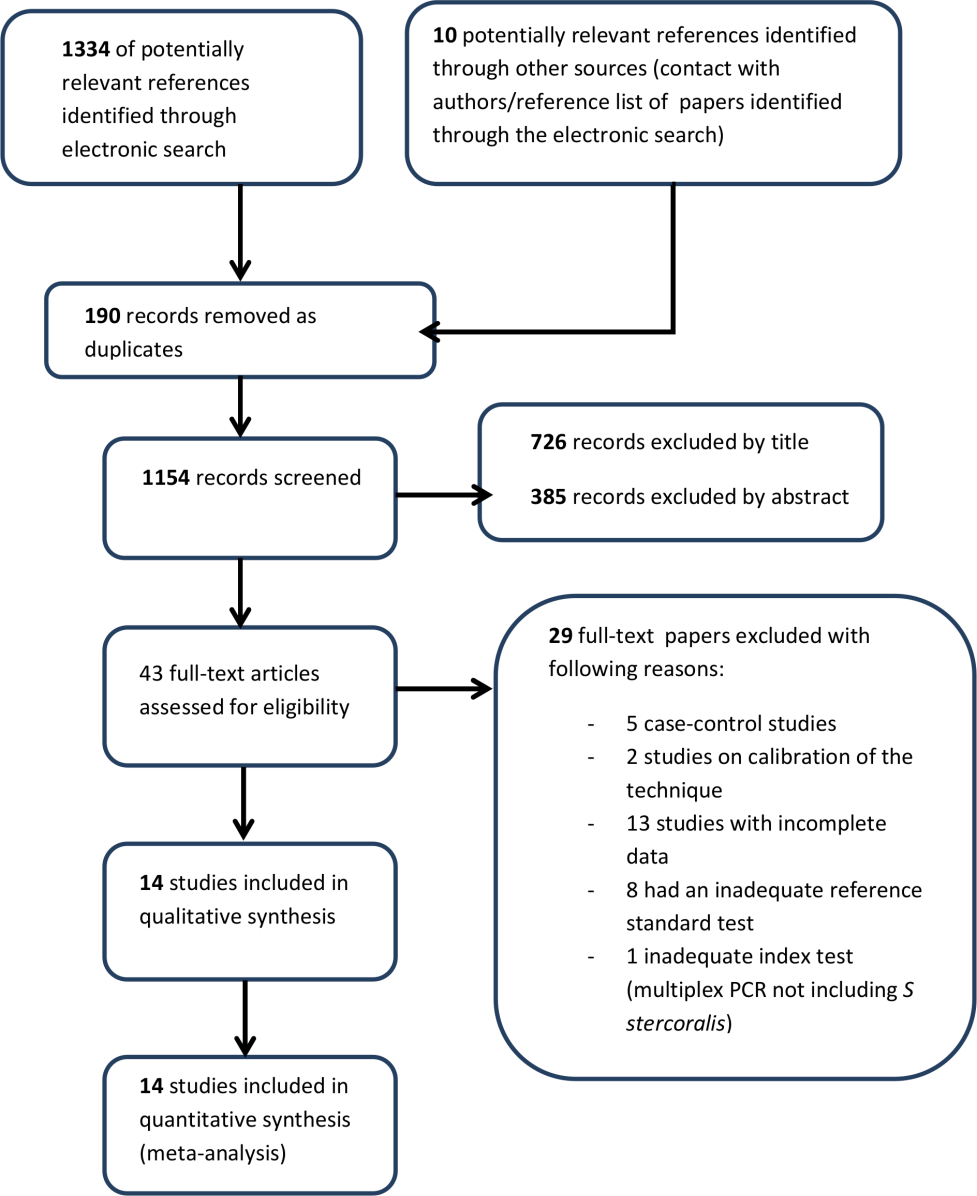
Study flow chart.

The electronic search identified 1334 records from the following databases: MEDLINE (448 records retrieved), Embase (516 records), CENTRAL (Cochrane library, 4 records), Lilacs (362 records); search on trial registries permitted to identify 4 further studies. The study flow is summarized in Fig 1.

Eventually, 14 studies were included both in quantitative and qualitative analyses. However, some studies evaluated either more than a single molecular method on the same pool of patients (in comparison to the same reference test) or a single molecular method on different subsets of patients (according to the results of different reference tests). In particular: two studies evaluated more than one molecular method (de Paula et al[17] tested both conventional and real-time PCR, Sharifdini et al [18] tested both nested and real-time PCR), one study [19] evaluated the same real-time PCR method performed in two different laboratories, and one study [20] evaluated the same index test (real-time PCR) both on patients positive to serology and on patients positive to APC. To handle and examine all these cases, we considered any experiment reported in a published paper as a separate study (Table 1).

**Table 1:** Main characteristics of the study sets included in the analysis.

Study	Index test(s) and PCR target	Reference test(s)	Setting	Population
Ahmad 2013[21]	Nested PCR ITS1 region of the rRNA genes	Serology	Endemic country	All ages
Amor 2016[22]	Real-time PCR 18S rRNA gene	Baermann	Endemic country	Children
Becker 2015_a [19]	Real-time PCR 18S rRNA gene	Combination of parasitological methods	Endemic country	All ages
Becker 2015_b[19]	Real-time PCR 18S rRNA gene	Combination of parasitological methods	Endemic country	All ages
Buonfrate 2017_a[20]	Real-time PCR 18S rRNA gene	APC	Non endemic country	Adults
Buonfrate 2017_b[20]	Real-time PCR 18S rRNA gene	Serology	Non endemic country	Adults
De Paula 2015_a [17]	Conventional 18S rRNA gene	APC	Endemic country	Not specified
De Paula 2015_b[17]	Real-time PCR 18S rRNA gene	APC	Endemic country	Not specified
Knopp 2014[11]	Real-time PCR 18S rRNA gene	Baermann	Endemic country	All ages
Lohd 2016[23]	Conventional PCR Specific interspersed repetitive sequence	Combination of parasitological methods	Endemic country	All ages
Meurs 2017[24]	Real-time PCR 18S rRNA gene	Combination of parasitological methods	Endemic country	All ages
Shar 2013[25]	Real-time PCR 18S rRNA gene	Combination of parasitological methods	Endemic country	Children
Sharifdini 2015_a[18]	Nested 18S rRNA gene	Combination of parasitological methods	Endemic country	Not specified
Sharifdini 2015_b[18]	Real-time PCR 18S rRNA gene	Combination of parasitological methods	Endemic country	Not specified
Sultana 2013[26]	Real-time PCR 18S rRNA gene	APC	Endemic country	Not specified
Ten Hove 2009[27]	Real-time PCR 18S rRNA gene	Baermann	Non endemic country	Adults
Verweij 2009[10]	Real-time PCR 18S rRNA gene	Combination of parasitological methods	Endemic country	All ages
Zueter 2014[28]	Real-time PCR 18S rRNA gene	Serology	Endemic country	Adults

ITS1 = Internal transcribed spacer 1

rRNA = ribosomal RNA

Therefore, 4 out of the 14 included studies generated more than one set of sensitivity and specificity estimates. Globally, the included studies comprised a total of 3060 participants (from 54 [21] to 466 [18]_individuals tested). Of note, 12 of 14 studies evaluated a real-time PCR technique, and all of them used the method described by Verweij *et a l*[10], which employs primers targeting the *S*. *stercoralis* 18S ribosomal RNA gene. A different target DNA was used in a couple of studies[21,23] only. Four studies evaluated either conventional[17,23] or nested[18,21] PCR. In addition, information on immunological status of the individuals tested was collected: only one study was conducted in immunocompromised patients[28]. Three studies compared PCR with serology. In a couple of cases the serology was a commercial ELISA test based on somatic antigens from *Strongyloides* L3 larvae[21,28], while the other study used an in-house IFAT based on intact *S*. *stercoralis* filariform larvae [20].

The information about the methods for the preservation of the biological samples is reported in supporting information table (S1 Table).The samples were mostly kept frozen or preserved in ethanol until DNA extraction. In a few studies, the samples were kept at room temperature or refrigerated, and processed within a short time. Only one study did not report the method for preserving the stool sample before the DNA extraction[28]. Another study protocol entailed the use of filter papers[23]. DNA extraction was performed with a commercial kit in almost all cases (S1 Table). Only Sharifdini *et al* [18] used an in-house method described previously[29]. The DNA extraction method was not reported in one case[28]. Most studies reported the use of controls for PCR inhibition (9 studies out of 14, S1 Table), and seven studies entailed the controls for DNA extraction. Neither PCR inhibition nor DNA extraction controls were reported by four studies. The validation of the PCR methods included the determination of a limit-of-detection (LOD) in four studies[17,18,23,26] only. Shar *et al[25]* reported the determination of LOD in the methods, but the value was not specified in the results.

**Fig 2:**
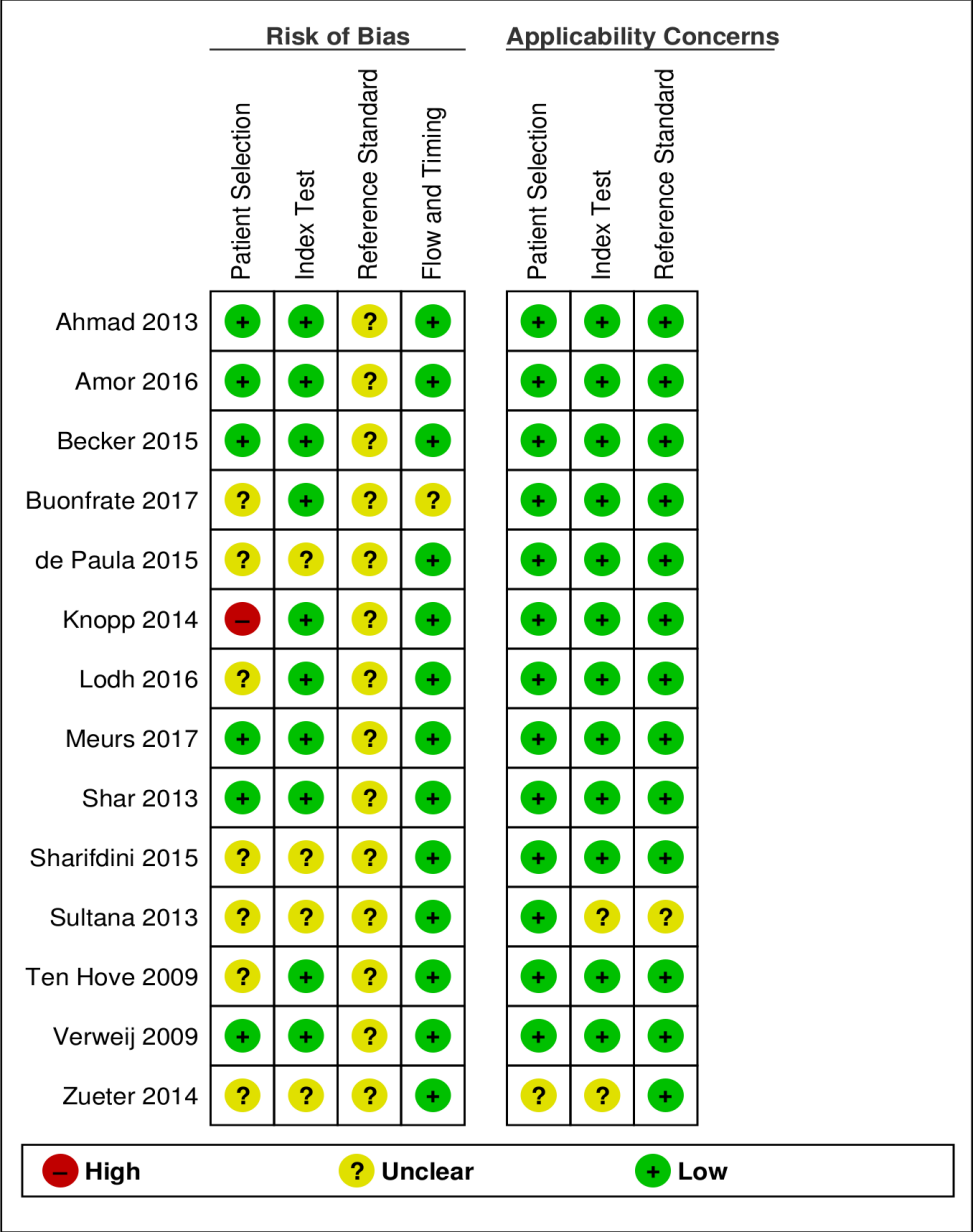
Risk of bias and applicability concerns summary.

**Fig 3:**
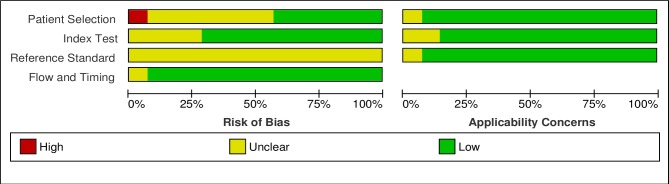
Risk of bias and applicability concerns graph.

Figs 2 and 3 show the results of the qualitative evaluation, in terms of rating for each included study and overall methodological quality, respectively.

**Fig 4:**
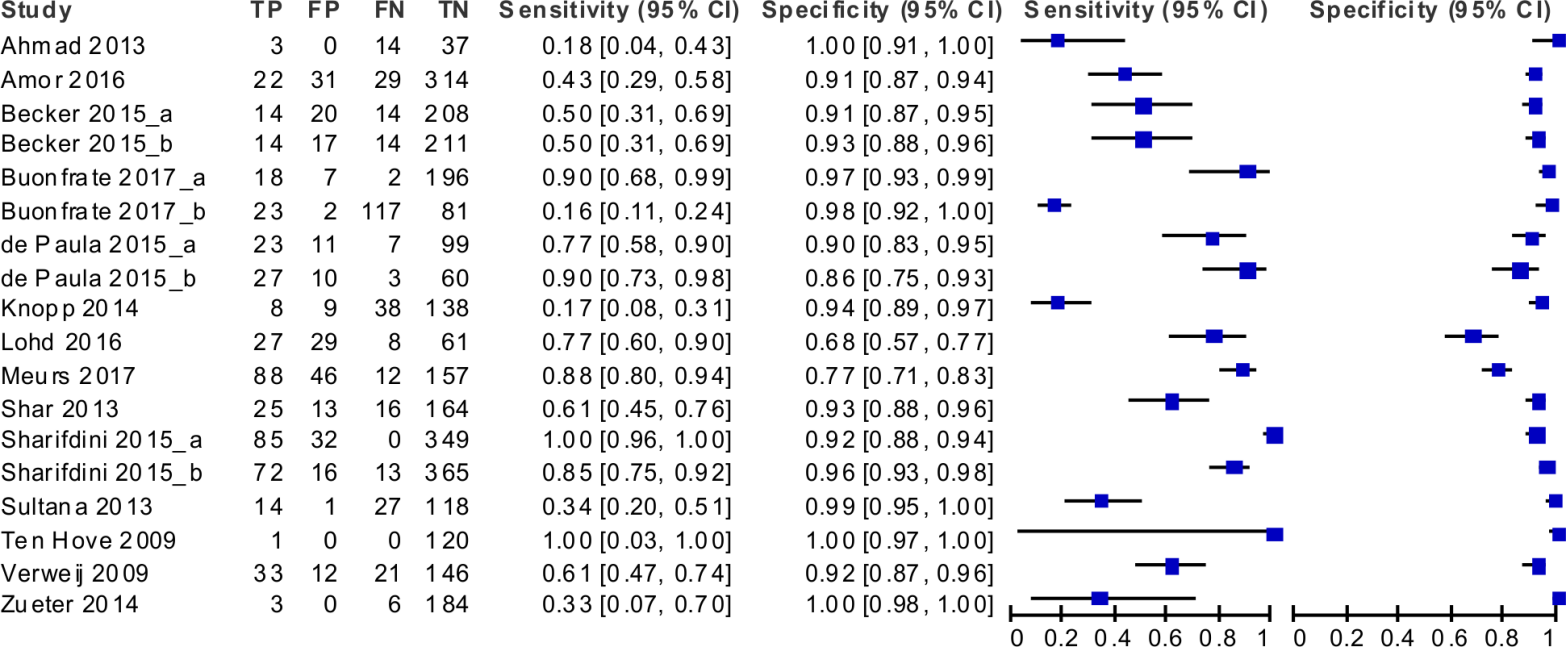
Forest plot showing the accuracy of the molecular techniques according to the single study sets.

As reported in the introduction, the evaluation of diagnostic tests for *S*. *stercoralis* is hampered by the lack of a gold standard. Therefore, the risk of bias associated to the reference test (possible incorrect classification) was assessed as unclear for all studies. Only two studies[11,20] applied any of the methods suggested for reporting diagnostic accuracy in absence of a gold standard[14]. In particular, Buonfrate et al[20] used a composite reference standard (CRS), while Knopp et al[11] applied a Bayesan latent class analysis (BLCA). Data from these studies were extracted, similarly to the other studies, in relation to the comparison of PCR to the other tests (without considering CRS or BLCM), in order to obtain a more homogenous evaluation of the index test. However, the results of CRS and BLCM were then compared to the global results of included studies. In the domain of the patient selection, the risk of bias was assessed as unclear for 7 studies. For 6 out of 7 studies, the reason was that the papers did not clearly report some relevant details about the patient sampling: whether the sampling was random or consecutive, or inappropriate exclusions were avoided. For one paper, the unclear risk was mainly due to the retrospective design of the study[20]. Finally, one paper clearly reported that patient sampling was not random, hence the risk of bias was assessed as high. [11] However, applicability concerns were assessed as low for all studies except one that evaluated the PCR accuracy in a cohort of cancer patients[28]. Fig 4 shows the accuracy reported in each study.

**Fig 5:**
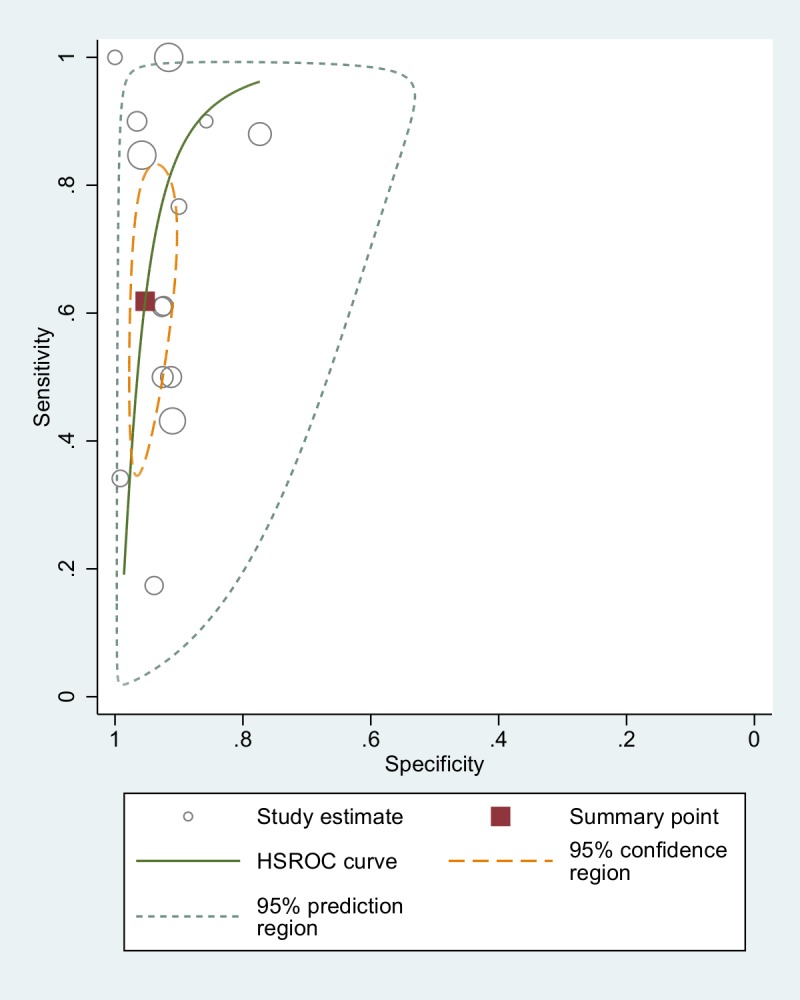
Accuracy of all PCR techniques (comparison with both fecal and serological tests): ROC curve.

The forest plot showed discrepancies in the results of the studies, particularly regarding sensitivity. As we included studies comparing PCR with different reference tests, this heterogeneity was partially expected. Nonetheless, we assessed the between-study variation in sensitivity and the degree of correlation between sensitivity and specificity by using the bivariate random effects approach introduced by Reitsma *et al*[14]. The variance of the logit of the sensitivity resulted 2.50 (95% CI: 1.12 to 5.49) and the correlation between logit of sensitivity and logit of specificity resulted -0.51 (95% CI: -0.82 to 0.02). Thus, we fitted a bivariate model to take into account heterogeneity as much as possible and to obtain pooled accuracy estimates of PCR versus all other techniques (Table 2). Globally, the accuracy of all PCR techniques resulted in a sensitivity of 61.8% (95% CI: 42.0 to 78.4) and a specificity of 95.2% (95% CI: 92.0 to 97.2). A visual summary of these findings, comprehensive of a confidence area of the estimates and a summary ROC curve obtained through hierarchical random effects approach (HSROC)[30] is displayed in Fig 5.

**Table 2:** Summary estimates of diagnostic accuracy of PCR techniques for the diagnosis of *Strongyloides stercoralis* infection.

Reference Test	All PCRs ^a^		Real Time PCR	
Serology or parasitological methods ^b^	Estimate (95% CI)	S.E.	Estimate (95% CI)	S.E.
Sensitivity	61.85% (42.0–78.4)	9.70	56.50% (39.2–72.4)	8.79
Specificity	95.27% (92.0–97.2)	1.28	95.38% (91.7–97.5)	1.40
DOR	32.7 (15.3–70.0)	12.6	26.8 (13.2–54.8)	9.77
LR+	13.1 (8.0–21.3)	3.2	12.2 (7.1–21.0)	3.38
LR-	0.40 (0.24–0.65)	0.09	0.45 (0.31–0.67)	0.08
1/LR-	2.5 (1.53–4.06)	0.62	2.2 (1.49–3.22)	0.43
**Parasitological methods** ^**b**^ **only**				
Sensitivity	71.76% (52.23–85.52)	8.72	64.42% (46.2–77.7)	8.31
Specificity	93.46% (90.35–95.61)	1.32	93.93% (90.3–96.3)	1.49
DOR	36.3 (15.4–85.4)	15.8	26.8 (12.7–56.6)	10.21
LR+	10.9 (7.2–16.6)	2.31	10.4 (6.4–16.8)	2.55
LR-	0.30 (0.16–0.55)	0.09	0.4 (0.25–0.60)	0.09
1/LR-	3.3 (1.49–3.22)	1.01	2.6 (1.66–3.98)	0.57

PCR, polymerase chain reaction; S.E., standard error; DOR, diagnostic odds ratio; LR, likelihood ratio

Estimates and S.Es for sensitivity and specificity are here reported in %.

^a^ Studies included conventional PCR, nested PCR, real-time PCR

^**b**^ Either Baermann method, agar plate culture, Harada-Mori culture, or a combination of fecal methods

When studies comparing PCR with serology-positive patients were excluded from analysis, the sensitivity resulted 71.8% (95% CI: 52.2 to 85.5), and specificity 93.4% (95% CI: 90.3 to 95.6). Real-time PCR techniques were then analyzed separately (Table 2), showing sensitivity and specificity values of 56.5% (95% CI 39–72) and 95.4% (95% CI 92–97), respectively.

**Fig 6:**
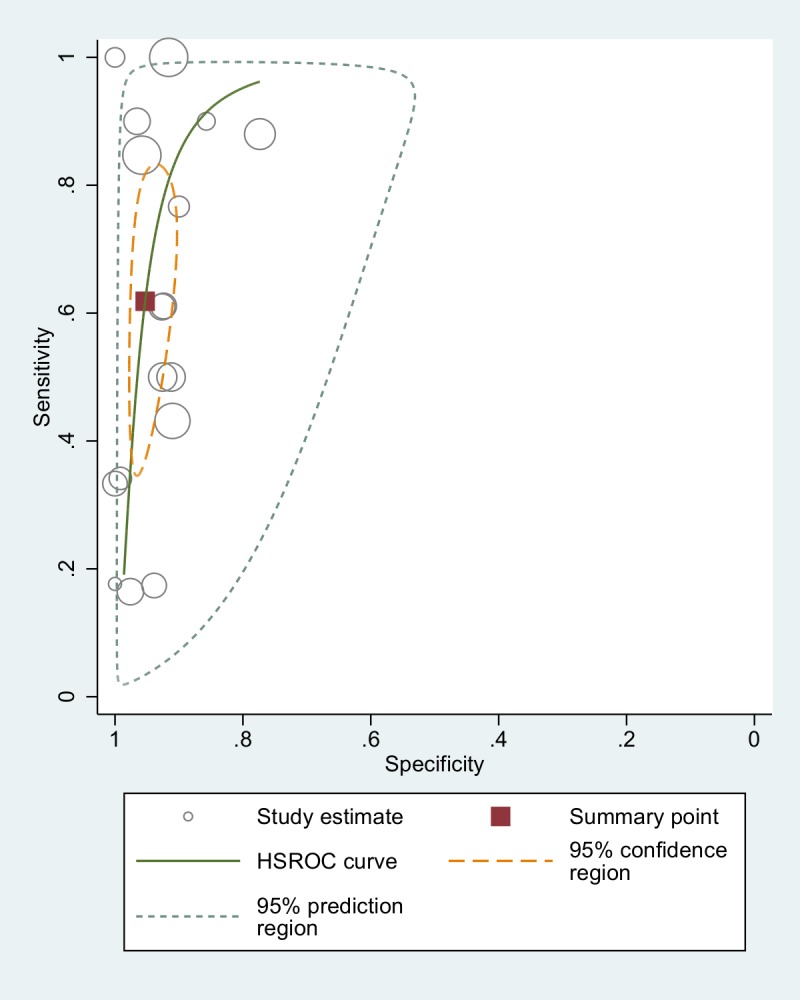
Accuracy of real-time PCR (comparison with fecal tests): ROC curve. Of note, the only study using a CRS (including serology) to assess the accuracy of real-time PCR demonstrated a sensitivity of 56.8%[20], which is almost the same value found with the meta-analysis. On the other hand, the only study using a Bayesian approach [11] demonstrated an extremely low sensitivity (11.6%) of real-time PCR.

Excluding serology from the analysis, the sensitivity and specificity values resulted 63.4% (95% CI 46–78) and 93.9% % (95% CI 90–96), respectively. The summary ROC curve is displayed in Fig 6.

## Discussion

Conventionally, PCR for *S*. *stercoralis* is considered 100% specific on the basis of the intrinsic characteristics of the technique. Although not confirming this value, the meta-analysis demonstrated a high specificity of PCR for the diagnosis of *S*. *stercoralis* infection, ranging from 93 to 95% according to the reference test. Moreover, it must be considered that the different reference standards used in the studies (implying that a sample PCR positive, but negative to all other fecal tests, is classified as a false positive) have most probably caused some underestimation of the specificity. On the other hand, the sensitivity resulted unsatisfactory, regardless of the reference test used: from 56% sensitivity when real-time PCR was compared to any other methods (including serology), to 71% when the results of any PCR techniques (either conventional, nested or real-time) were compared to fecal methods only. One possible explanation for this low sensitivity, particularly when compared with serological tests, is the irregular larval output observed in chronic strongyloidiasis. Therefore, PCR techniques might face the same problem as the conventional parasitological techniques. As a matter of fact, PCR has not proven to be diagnostically superior to other parasitological techniques such as the Baermann method or APC, particularly in low-density infections where the larval output is low and irregular [5]. Moreover, one cause of the low sensitivity of PCR might be the small quantity of fecal sample analyzed [31], particularly relevant when the larvae are scarcely shed in feces.

Unfortunately, only a few included studies assessed the LOD of their techniques, that could permit a more accurate evaluation of the sensitivity of the PCR in relation to different levels of larval shedding. This information would be useful also to compare different techniques used in different studies, and should be better reported.

On the other hand, the sample preservation methods were reported by all but one authors of the included studies: they were all adequate, and presumably did not affect the results of the PCR. Also, DNA extraction was almost always conducted with commercial kits based on silica-membrane-DNA purification. All the automated methods used were highly reliable and the studies resulted homogeneous in relation to this aspect. Only one study reported an in-house method for DNA isolation that implies an organic solvent extraction and alcohol precipitation.

One reason for the low sensitivity might be represented by the presence of PCR inhibitors, commonly found in fecal samples. In fact, some authors did not report the use of controls for PCR inhibition. Knopp *et al*[11], who found the lowest sensitivity value of real-time PCR (when not considering the studies comparing PCR with serology) declared that the absence of controls for PCR inhibition was one of the limitations of their study. Therefore, we cannot exclude that PCR inhibition occurred and affected the results of some studies. However, most included papers reported the use of controls for PCR inhibition, and sensitivity resulted variable and seldom achieved 90%. In any case, these controls are of primary importance to confirm the correct execution of the PCR, and are therefore recommended both in research studies and in routine practice.

Analogously, the use of controls for DNA extraction was not reported by all authors, and it cannot be ruled out that a low efficiency in DNA extraction affected the results of PCR. Also in this case, the use of such controls is recommended both in routine and in research activities.

The interpretation of the results from a clinical point of view is resumed in the summary of findings table (Table 3).

**Table 3:** Summary of findings table for the review of PCR techniques for the diagnosis of *Strongyloides stercoralis* infection.

Interpretative criteria to define: Index vs. Reference Test	Effect (95% CI)	Number of studies	Mean Prevalence (95% CI)	What do these results mean?
All PCR^a^ vs. Serology or parasitological methods^b^	Sensitivity: 61.8% (42.0–78.4) Specificity: 95.3% (92.0–97.2)	17	21.1% (13.8 to 28.4)	Assuming (based on the mean prevalence) 21 out of 100 patients with SSI, eight would be missed by a single PCR test (38% of 21). Of the 79 patients without SSI, four (5%) would have a false positive result of the PCR test.
Al PCR vs. parasitological methods only	Sensitivity: 71.8% (52.2–85.5) Specificity: 93.5% (90.3–95.6)	14	18.5% (13.4 to 23.6)	Assuming 18 out of 100 patients with SSI, five would be missed by a single PCR test. Of the 82 patients without SSI, five would have a false positive result of the PCR test.
Real-time PCR vs. Serology or parasitological methods	Sensitivity: 56.5% (39.2–72.4) Specificity: 95.4% (91.7–97.5)	14	20.5% (11.6 to 29.4)	Assuming 20 out of 100 patients with SSI, nine would be missed by a single PCR test. Of the other 80, four will have a false positive result of the PCR test.
Real-time PCR vs. parasitological methods only	Sensitivity: 64.4% (46.2–77.7) Specificity: 93.9% (90.3–96.3)	12	20.3% (9.9 to 30.8)	Assuming 20 out of 100 patients with SSI, seven would be missed by a single PCR test. Of the other 80, five would have a false positive result of the PCR test.

PCR, polymerase chain reaction; SSI, *S*. *stercoralis* infection. Estimates for sensitivity and specificity are here reported in %.

^**a**^ Studies included conventional PCR, nested PCR, real-time PCR

^b^ Either Baermann method, agar plate culture, Harada-Mori culture, or a combination of fecal methods

Indeed, PCR is not adequate for universal screening of strongyloidiasis, as it would entail an excessive risk of missing diagnoses of a potentially fatal infection. It could rather be a valid option as a confirmatory test in case of positive serology. Moreover, it could be used as an alternative to other fecal-based tests for the screening of immunosuppressed patients, for whom the sensitivity of serology decreases[32]. However, also in this latter group it should be used in addition to serology, in order to increase case-detection in these patients particularly at risk of developing severe infection.

Unfortunately, as it results from the qualitative evaluation of the included studies, we suggest that the lack of a gold standard for the diagnosis may hamper the results of diagnostic studies. This problem is frequently encountered in parasitology. The comparison of PCR with the fecal methods which proved to be sufficiently sensitive for the diagnosis of strongyloidiasis (namely, Baermann and APC) could be seen as the best option to validate the accuracy of PCR, as they all rely on larval shedding, indicating the presence of active infection. However, the sensitivity of Baermann and APC is still inadequately low to safely rule out the infection, when resulting negative. For this reason, using them as reference tests tends to result in an overestimation of the sensitivity of PCR. Serology detects the antibodies against larval antigens, hence it does not rely on the presence of larvae in stool, that is often inconstant. Despite the possibility of false positive results (as reported in the introduction), we decided to add the comparison with serology to highlight that the sensitivity of PCR is presumably lower than that found when compared with the other fecal methods.

Although methods to assess the test accuracy in the absence of a gold standard have been proposed [14], they are seldom applied, as it resulted from our review, too (only a couple of studies proposed an alternative model for the classification of the results). Indeed, our investigation highlighted that, in absence of a validated reference standard, different studies considered different reference tests for the evaluation of the accuracy of PCR, leading to difficulties in the direct comparison of the results.

Another limitation of our study is that it was not possible to analyze the influence of setting and age on the accuracy, because of the relatively low number of studies included in the meta-analysis. Due to the distinct pools of patients (defined through the different reference tests) of the PCR experiments included in the analysis, a certain degree of heterogeneity was inevitably expected. Indeed, the measure of correlation between sensitivity and specificity provided evidence of a heterogeneity that should not be ignored. This heterogeneity may be also largely caused by variations between tests in terms of country setting, population age or by a threshold effect. Nonetheless, the utilization of statistical techniques that take this heterogeneity into account for the estimation of summary measures, such as the bivariate model by Reitsma *et al* [14], allowed for exhaustive and robust estimates as shown in Table 2. As the number of studies included did not allow for a proper analysis of all possible sub-cases of index-reference tests, these estimates shall be considered as pooled accuracy measures of the PCR techniques versus all other techniques.

### Conclusions

In summary, the results of this review suggest that, although the PCR technique is highly specific, it should not yet be recommended for universal screening, nor as a stand-alone method for the individual diagnosis of *S*. *stercoralis* infection. However, PCR has a role as a confirmatory test. Additional studies investigating the accuracy of this and other diagnostic tests for this infection, using appropriate methods to cope with the absence of a gold standard, are needed to improve the screening and management of this neglected infection.

## Supporting information

S1 Fig PRISMA checklist. (DOCX)

S2 Fig Search strategy for PubMed. (DOCX)

S1 Table Methods reported by included papers for preservation of the samples, DNA extraction and use of controls for DNA extraction/PCR inhibition. (DOCX)

## References

[1] Bisoffi Z, Buonfrate D, Montresor A, Requena-Mendez A, Munoz J, et al (2013) *Strongyloides stercoralis*: a plea for action. PLoS Negl Trop Dis 7: e2214 doi: 10.1371/journal.pntd.0002214 23675546 10.1371/journal.pntd.0002214 PMC3649953

[2] Greaves D, Coggle S, Pollard C, Aliyu SH, Moore EM (2013) *Strongyloides stercoralis* infection. BMJ 347: f4610 doi: 10.1136/bmj.f4610 23900531 10.1136/bmj.f4610

[3] Henriquez-Camacho C, Gotuzzo E, Echevarria J, White AC Jr., Terashima A, et al (2016) Ivermectin versus albendazole or thiabendazole for *Strongyloides stercoralis* infection. Cochrane Database Syst Rev: CD007745 doi: 10.1002/14651858.CD007745.pub3 26778150 10.1002/14651858.CD007745.pub3 PMC4916931

[4] Mejia R, Nutman TB (2012) Screening, prevention, and treatment for hyperinfection syndrome and disseminated infections caused by *Strongyloides stercoralis*. Curr Opin Infect Dis 25: 458–463. doi: 10.1097/QCO.0b013e3283551dbd 22691685 10.1097/QCO.0b013e3283551dbd PMC3430846

[5] Requena-Mendez A, Chiodini P, Bisoffi Z, Buonfrate D, Gotuzzo E, et al (2013) The laboratory diagnosis and follow up of strongyloidiasis: a systematic review. PLoS Negl Trop Dis 7: e2002 doi: 10.1371/journal.pntd.0002002 23350004 10.1371/journal.pntd.0002002 PMC3547839

[6] Campo Polanco L, Gutierrez LA, Cardona Arias J (2014) [Diagnosis of *Strongyloides Stercoralis* infection: meta-analysis on evaluation of conventional parasitological methods (1980–2013)]. Rev Esp Salud Publica 88: 581–600. doi: 10.4321/S1135-57272014000500004 25327268 10.4321/S1135-57272014000500004

[7] Bisoffi Z, Buonfrate D, Sequi M, Mejia R, Cimino RO, et al (2014) Diagnostic accuracy of five serologic tests for *Strongyloides stercoralis* infection. PLoS Negl Trop Dis 8: e2640 doi: 10.1371/journal.pntd.0002640 24427320 10.1371/journal.pntd.0002640 PMC3890421

[8] Ramanathan R, Burbelo PD, Groot S, Iadarola MJ, Neva FA, et al (2008) A luciferase immunoprecipitation systems assay enhances the sensitivity and specificity of diagnosis of *Strongyloides stercoralis* infection. J Infect Dis 198: 444–451. doi: 10.1086/589718 18558872 10.1086/589718 PMC3379004

[9] Krolewiecki AJ, Ramanathan R, Fink V, McAuliffe I, Cajal SP, et al (2010) Improved diagnosis of *Strongyloides stercoralis* using recombinant antigen-based serologies in a community-wide study in northern Argentina. Clin Vaccine Immunol 17: 1624–1630. doi: 10.1128/CVI.00259-10 20739501 10.1128/CVI.00259-10 PMC2952987

[10] Verweij JJ, Canales M, Polman K, Ziem J, Brienen EA, et al (2009) Molecular diagnosis of *Strongyloides stercoralis* in faecal samples using real-time PCR. Trans R Soc Trop Med Hyg 103: 342–346. doi: 10.1016/j.trstmh.2008.12.001 19195671 10.1016/j.trstmh.2008.12.001

[11] Knopp S, Salim N, Schindler T, Karagiannis Voules DA, Rothen J, et al (2014) Diagnostic accuracy of Kato-Katz, FLOTAC, Baermann, and PCR methods for the detection of light-intensity hookworm and *Strongyloides stercoralis* infections in Tanzania. Am J Trop Med Hyg 90: 535–545. doi: 10.4269/ajtmh.13-0268 24445211 10.4269/ajtmh.13-0268 PMC3945701

[12] Whiting PF, Rutjes Aw Fau—Westwood ME, Westwood Me Fau—Mallett S, Mallett S Fau—Deeks JJ, Deeks Jj Fau—Reitsma JB, et al. QUADAS-2: a revised tool for the quality assessment of diagnostic accuracy studies. 10.7326/0003-4819-155-8-201110180-00009 22007046

[13] http://community.cochrane.org/tools/review-production-tools/revman-5.

[14] Reitsma JB, Glas AS, Rutjes AW, Scholten RJ, Bossuyt PM, et al (2005) Bivariate analysis of sensitivity and specificity produces informative summary measures in diagnostic reviews. J Clin Epidemiol 58: 982–990. doi: 10.1016/j.jclinepi.2005.02.022 16168343 10.1016/j.jclinepi.2005.02.022

[15] Rogan WJ, Gladen B (1978) Estimating prevalence from the results of a screening test. Am J Epidemiol 107: 71–76. 623091 10.1093/oxfordjournals.aje.a112510

[16] Rutter CM, Gatsonis CA (2001) A hierarchical regression approach to meta-analysis of diagnostic test accuracy evaluations. Stat Med 20: 2865–2884. 11568945 10.1002/sim.942

[17] Paula FM, Malta Fde M, Marques PD, Sitta RB, Pinho JR, et al (2015) Molecular diagnosis of strongyloidiasis in tropical areas: a comparison of conventional and real-time polymerase chain reaction with parasitological methods. Mem Inst Oswaldo Cruz 110: 272–274. doi: 10.1590/0074-02760140371 25946255 10.1590/0074-02760140371 PMC4489462

[18] Sharifdini M, Mirhendi H, Ashrafi K, Hosseini M, Mohebali M, et al (2015) Comparison of Nested Polymerase Chain Reaction and Real-Time Polymerase Chain Reaction with Parasitological Methods for Detection of *Strongyloides stercoralis* in Human Fecal Samples. Am J Trop Med Hyg 93: 1285–1291. doi: 10.4269/ajtmh.15-0309 26350449 10.4269/ajtmh.15-0309 PMC4674247

[19] Becker SL, Piraisoody N, Kramme S, Marti H, Silue KD, et al (2015) Real-time PCR for detection of *Strongyloides stercoralis* in human stool samples from Cote d'Ivoire: diagnostic accuracy, inter-laboratory comparison and patterns of hookworm co-infection. Acta Trop 150: 210–217. doi: 10.1016/j.actatropica.2015.07.019 26215130 10.1016/j.actatropica.2015.07.019

[20] Buonfrate D, Perandin F, Formenti F, Bisoffi Z (2017) A retrospective study comparing agar plate culture, indirect immunofluorescence and real-time PCR for the diagnosis of *Strongyloides stercoralis* infection. Parasitology: 1–5. 10.1017/S0031182016002559 28073382

[21] Ahmad AF, Hadip F, Ngui R, Lim YA, Mahmud R (2013) Serological and molecular detection of *Strongyloides stercoralis* infection among an Orang Asli community in Malaysia. Parasitol Res 112: 2811–2816. doi: 10.1007/s00436-013-3450-z 23666229 10.1007/s00436-013-3450-z

[22] Amor A, Rodriguez E, Saugar JM, Arroyo A, Lopez-Quintana B, et al (2016) High prevalence of *Strongyloides stercoralis* in school-aged children in a rural highland of north-western Ethiopia: the role of intensive diagnostic work-up. Parasit Vectors 9: 617 doi: 10.1186/s13071-016-1912-8 27903301 10.1186/s13071-016-1912-8 PMC5131444

[23] Lodh N, Caro R, Sofer S, Scott A, Krolewiecki A, et al (2016) Diagnosis of *Strongyloides stercoralis*: Detection of parasite-derived DNA in urine. Acta Trop 163: 9–13. doi: 10.1016/j.actatropica.2016.07.014 27456935 10.1016/j.actatropica.2016.07.014 PMC5117362

[24] Meurs L, Polderman AM, Vinkeles Melchers NV, Brienen EA, Verweij JJ, et al (2017) Diagnosing Polyparasitism in a High-Prevalence Setting in Beira, Mozambique: Detection of Intestinal Parasites in Fecal Samples by Microscopy and Real-Time PCR. PLoS Negl Trop Dis 11: e0005310 doi: 10.1371/journal.pntd.0005310 28114314 10.1371/journal.pntd.0005310 PMC5289637

[25] Schar F, Odermatt P, Khieu V, Panning M, Duong S, et al (2013) Evaluation of real-time PCR for *Strongyloides stercoralis* and hookworm as diagnostic tool in asymptomatic schoolchildren in Cambodia. Acta Trop 126: 89–92. doi: 10.1016/j.actatropica.2012.12.012 23298731 10.1016/j.actatropica.2012.12.012

[26] Sultana Y, Jeoffreys N, Watts MR, Gilbert GL, Lee R (2013) Real-time polymerase chain reaction for detection of *Strongyloides stercoralis* in stool. Am J Trop Med Hyg 88: 1048–1051. doi: 10.4269/ajtmh.12-0437 23568289 10.4269/ajtmh.12-0437 PMC3752801

[27] ten Hove RJ, van Esbroeck M Fau—Vervoort T, Vervoort T Fau—van den Ende J, van den Ende J Fau—van Lieshout L, van Lieshout L Fau—Verweij JJ, et al. Molecular diagnostics of intestinal parasites in returning travellers. 10.1007/s10096-009-0745-1 PMC2758195 19415354

[28] Zueter AM, Mohamed Z, Abdullah AD, Mohamad N, Arifin N, et al (2014) Detection of *Strongyloides stercoralis* infection among cancer patients in a major hospital in Kelantan, Malaysia. Singapore Med J 55: 367–371. doi: 10.11622/smedj.2014088 25091885 10.11622/smedj.2014088 PMC4291962

[29] Repetto SA, Alba Soto Cd Fau—Cazorla SI, Cazorla Si Fau—Tayeldin ML, Tayeldin Ml Fau—Cuello S, Cuello S Fau—Lasala MB, et al. An improved DNA isolation technique for PCR detection of *Strongyloides stercoralis* in stool samples. 10.1016/j.actatropica.2013.02.003 23416126

[30] Rutjes AW, Reitsma JB, Coomarasamy A, Khan KS, Bossuyt PM (2007) Evaluation of diagnostic tests when there is no gold standard. A review of methods. Health Technol Assess 11: iii, ix-51. 10.3310/hta11500 18021577

[31] Requena-Mendez A, Buonfrate D, Bisoffi Z, Munoz J (2014) Advances in the Diagnosis of Human Strongyloidiasis. Curr Trop Med Rep 1: 207–215.

[32] Luvira V, Trakulhun K, Mungthin M, Naaglor T, Chantawat N, et al (2016) Comparative Diagnosis of Strongyloidiasis in Immunocompromised Patients. Am J Trop Med Hyg 95: 401–404. doi: 10.4269/ajtmh.16-0068 27296387 10.4269/ajtmh.16-0068 PMC4973189

