# Supplementary material for: Accuracy of molecular biology techniques for the diagnosis of Strongyloides stercoralis infection—A systematic review and meta-analysis
Source: PLoS Negl Trop Dis. 2018 Feb 9;12(2):e0006229. doi: 10.1371/journal.pntd.0006229 (PMC5823464; doi:10.1371/journal.pntd.0006229)
Supplement: S2 Fig — (DOCX) [file pntd.0006229.s002.docx]

**((((((((((((((("Strongyloidiasis"[Mesh]) OR "Strongyloides"[Mesh]) OR "Strongyloides stercoralis"[Mesh])) OR "strongyloides stercoralis"[Title/Abstract]) OR "strongyloides"[Title/Abstract]) OR strongyloid*[Title/Abstract]) OR "nematode"[Title/Abstract]) OR nematod*[Title/Abstract])) OR "soil helminths"[Title/Abstract]))) OR "anguillulose"[Title/Abstract]) OR "anguillulosis"[Title/Abstract]) OR anguillulos*[Title/Abstract]**

**AND**

**((((((((((((((((("Polymerase Chain Reaction"[Mesh:noexp]) OR "Real-Time Polymerase Chain Reaction"[Mesh]) OR "Multiplex Polymerase Chain Reaction"[Mesh]))))) OR (((((((((((((((((((((((((((((("polymerase chain reaction"[Title/Abstract]) OR "polymerase chain reaction pcr"[Title/Abstract]) OR "polymerase chain reaction (PCR)"[Title/Abstract]) OR "pcr"[Title/Abstract]) OR "real time polymerase chain reaction"[Title/Abstract]) OR Real-Time Polymerase Chain Reaction[Title/Abstract]) OR "real time pcr"[Title/Abstract]) OR "Real-time PCR"[Title/Abstract]) OR "real time quantitative polymerase chain reaction"[Title/Abstract]) OR "real time quantitative pcr"[Title/Abstract]) OR "real-time quantitative polymerase chain reaction"[Title/Abstract]) OR "real-time quantitative pcr"[Title/Abstract]) OR "reverse transcription polymerase chain reaction rt pcr"[Title/Abstract]) OR "real time rt pcr"[Title/Abstract]) OR "multiplex polymerase chain reaction"[Title/Abstract]) OR "multiplex polymerase chain reaction pcr"[Title/Abstract]) OR "multiplex pcr"[Title/Abstract]) OR "multiplex real time polymerase chain reaction"[Title/Abstract]) OR "multiplex real-time polymerase chain reaction"[Title/Abstract]) OR "multiplex real time pcr"[Title/Abstract]) OR "multiplex real-time pcr"[Title/Abstract]) OR "multiplex rt pcr"[Title/Abstract]) OR "multiplex reverse transcription polymerase chain reaction"[Title/Abstract]) OR "quantitative real time polymerase chain reaction qpcr"[Title/Abstract]) OR "quantitative real-time polymerase chain reaction qpcr"[Title/Abstract]) OR "loop mediated isothermal amplification lamp"[Title/Abstract]) OR "loop-mediated isothermal amplification lamp"[Title/Abstract]) OR "lamp"[Title/Abstract]) OR "reverse transcription loop mediated isothermal amplification rt lamp"[Title/Abstract]) OR "quantitative loop-mediated isothermal amplification (qLAMP)"[Title/Abstract])**
