# Supplementary material for: Accuracy of molecular biology techniques for the diagnosis of Strongyloides stercoralis infection—A systematic review and meta-analysis
Source: PLoS Negl Trop Dis. 2018 Feb 9;12(2):e0006229. doi: 10.1371/journal.pntd.0006229 (PMC5823464; doi:10.1371/journal.pntd.0006229)
Supplement: S1 Table — (DOCX) [file pntd.0006229.s003.docx]

| **Paper** | **Preservation of the samples** | **DNA extraction method** | **Controls for DNA extraction/PCR inhibition** |
| --- | --- | --- | --- |
| Ahmad | Samples at 2-8°C if processed within 48 hours, alternatively kept at -20° for longer storage | Mo Bio Power Soil DNA isolation kit | Not specified |
| Amor | Kept at room temperature and processed within 4 hours | QIAamp DNA stool mini kit (Qiagen, Hilden, Germany) | Control of PCR inhibition |
| Becker | Preserved in 70% ethanol | QIAamp DNA stool mini kit (Qiagen, Hilden, Germany) | Not specified |
| Buonfrate | Samples kept at -20°C | Magnapure LC.2 Roche | Controls for both DNA extraction and PCR inhibition |
| De Paula | Preserved in 70% ethanol | QIAamp DNA stool mini kit (Qiagen, Hilden, Germany) | Controls for both DNA extraction and PCR inhibition. |
| Knopp | Stored at -20°C | QIAamp Tissue kit spin columns (QIAgen, Hilden, Germany) | Only for DNA extraction. |
| Lodh | Dried filter disc | QIAmpDNA Blood Mini kit (Qiagen, MD) | Not specified**.** |
| Meurs | Preserved in 96% ethanol for transport to the lab, where they were kept at -20°C | QIAamp Spin Columns/Mini kit (Qiagen, Germany) | Controls for both DNA extraction and PCR inhibition |
| Shar | Stored at -20°C | QIAamp DNA stool mini kit (Qiagen, Hilden, Germany) | Control PCR inhibition. |
| Sharifdini | Preserved in 70% ethanol | Phenol/chloroform/isoamyl alchohol extraction and isopropanol precipitation | Not specified. |
| Sultana | Stored at -20°C | Power soil kit | Control PCR inhibition. |
| Ten Hove | Immediately processed | QIAamp Tissue kit spin columns (QIAgen, Hilden, Germany) | Controls for both DNA extraction and PCR inhibition |
| Verweij | Either frozen or suspended in ethanol | QIAamp Tissue kit spin columns (QIAgen, Hilden, Germany) | Controls for both DNA extraction and PCR inhibition |
| Zueter | Not specified | Not specified | Controls for both DNA extraction and PCR inhibition |
